# Epidemiology of traumatic falls after Hurricane Maria in Puerto Rico

**DOI:** 10.1186/s40621-020-00236-3

**Published:** 2020-06-01

**Authors:** Laura Ramírez-Martínez, Mariella Chamah-Nicolás, Mariely Nieves-Plaza, Javier Ruiz-Rodríguez, Pedro Ruiz-Medina, Ediel O. Ramos-Melendez, Pablo Rodríguez-Ortiz

**Affiliations:** 1 Puerto Rico Trauma Hospital, PO Box 2129, San Juan, 00922 Puerto Rico; 2 Department of Surgery, Trauma Research Program, Medical Sciences Campus, University of Puerto Rico, PO Box 365067, San Juan, 00967 Puerto Rico

**Keywords:** Falls, Injury, Fall-related injuries, Trauma, Hurricane, Natural disasters

## Abstract

**Background:**

Hurricanes are among the most devastating natural disasters, playing a significant role in public health. Currently, the epidemiology of fall-related injuries after the occurrence of a tropical storm is not well described. This study aims to compare the demographical patterns, clinical profile, hospital course, and costs of patients admitted to the Puerto Rico Trauma Hospital before and after Hurricane Maria.

**Methods:**

A retrospective study was performed to compare fall-related injuries after the hurricane (September 20, 2017 - January 20, 2018) with a control period (same period in 2014–2016). Comparison between the groups was done using chi-square, Mann-Whitney test, and logistical regression.

**Results:**

After the hurricane, there was an increase in the proportion of fall-related admissions in subjects aged 40–64 years (39.2% vs. 50.6%) and a decrease among those aged 18–39 years (16.0% vs. 5.9%), when compared with the previous years. A greater proportion of patients presented with work related injuries (3.9% vs. 9.4%). No significant differences were identified for sex, Glasgow Coma Scale, Injury Severity Score, and hospital outcomes (hospital and intensive care unit days, mechanical ventilation, and mortality). Intracranial injuries were marginally higher post-Maria (*p* = 0.06). In multivariate analysis, during the post-Maria period, an increased risk of fall-related injuries was observed among subjects ≥40 years (OR: 3.20) and injuries related to recovery work (OR: 2.64) (*p* < 0.05).

**Conclusions:**

Our study shows that there is an increased risk of fall-related injuries among middle-aged individuals after a hurricane, causing significant changes in epidemiology. This study helps to elucidate the health consequences of falls and, in doing so, improves healthcare preparedness, interventions, and planning for future natural disasters.

## Background

Hurricanes are among the most devastating natural disasters, playing a significant role in public health due to its effects on injury, illness, mental disorders, and mortality (Binder and Sanderson 1987; Malilay et al. 2014; Uscher-Pines et al. 2009). In particular, the period after a natural disaster represents a serious threat for injury, as hazardous roads along with delayed cleanup and rebuilding processes place civilians in harm’s way (Malilay et al. 2014; Uscher-Pines et al. 2009; Curran et al. 2017; Adams et al. 2011; Al-Rousan et al. 2014; Berry and Miller 2008). Lack of electricity, slow and short periods of assistance, and prolonged home displacement all provide conditions that increase the likelihood for injuries (Uscher-Pines et al. 2009; Curran et al. 2017; Adams et al. 2011; Al-Rousan et al. 2014). It is prior to storm impact, during the catastrophe and recovery phase when falls are especially prevalent and, as a result, they constitute the main mechanism of injury (Curran et al. 2017; Sullivent et al. 2006; Frasqueri-Quintana et al. 2019; Shulyz et al. 2005). Fall-related injuries are normally associated with an increased use of healthcare services and represent a significant source of morbidity and mortality among the elderly (Berry and Miller 2008; Sullivent et al. 2006; Frasqueri-Quintana et al. 2019; Bergen et al. 2014; Hefny et al. 2016; Rubenstein 2006). However, after a hurricane, there is a shift in previously known epidemiologic trends, as falls in men and middle-aged individuals become the prevalent demographic group (Malilay et al. 2014; Uscher-Pines et al. 2009).

Environmental factors account for approximately 30–50% of all fall injuries. Imbalance, multiple medications, previous falls, advanced age, gender, visual impairment, and cognitive deterioration compose the remaining percentage (Rubenstein 2006; Alamgir et al. 2015; Florence et al. 2018; Lee et al. 2013). Falls can significantly impact an individual’s quality of life and independence, while also having substantial medical and social costs. In 2015, it was estimated that the United States’ medical costs for falls totaled nearly $50 billion (Burns and Kakara 2018; Florence et al. 2018). However, no study have evaluated health care costs of falls in Puerto Rico. The combination of a rapidly aging population and the commonality of natural disasters have made falls a greater public health concern (Granhed et al. 2017).

Not every person that suffers a fall seeks medical attention and the majority do not even require medical intervention. However, of the fall victims that do seek medical attention, 40–70% sustain significant traumatic injuries. After a fall, common injuries include bone fractures, head injuries, intrathoracic and intra-abdominal wounds, bruises, and sprains (Berry and Miller 2008; Bergen et al. 2014; Hefny et al. 2016; Rubenstein 2006; Alamgir et al. 2015). Fractures to the extremities, after a fall, are also well characterized and can vary depending on the fall height. For instance, at same level falls, upper extremity fractures are common, but at higher falls, fractures tend to localize in the lower extremities. Additionally, spine injuries, predominantly in the lumbar region, range from 13% in mixed falls to 36% in high falls. High altitude falls also produce more thoracic injuries, rib fractures, lung contusions, pneumothorax, hemothorax, and cardiac and aortic ruptures (Granhed et al. 2017).

Hurricane Maria, a deadly Category 4 tropical cyclone made landfall in Puerto Rico (PR) on September 20, 2017, 2 weeks after Hurricane Irma. As of today, it is regarded as the worst natural disaster to affect the island. All 3.4 million inhabitants were left immediately without electricity as the island’s power grid was completely destroyed, leading to the worst electrical blackout in US history (Pasch et al. 2017; Houser and Marsters 2018). The entire island experienced widespread catastrophic destruction. After the hurricane, PR endured a slow recovery process, which directly translated into extended periods of home displacements, treacherous roads, structural damage, an island-wide communication blackout, and a shortage of food, water, and gas (Milken Institute School of Public Health at The George Washington University 2018; Kishore et al. 2018). The lack of the aforementioned basic resources led to a major humanitarian crisis.

Early data provided by the government of Puerto Rico suggested that the fatality toll related to the passage of Hurricane Maria totaled 64 deaths. However, it has been found through several studies which have attempted to asses Maria’s impact on mortality that approximately 2975 to 4645 people died as a result of the hurricane (Milken Institute School of Public Health at The George Washington University 2018; Kishore et al. 2018; Santos-Lozada and Howard 2018; Santos-Burgoa et al. 2018). These studies found a significant excess mortality among older adults and men after the hurricane (Santos-Burgoa et al. 2018; Cruz-Cano and Mead 2019). No study, however, has explored the impact of this weather phenomenon on traumatic injuries. The Puerto Rico Trauma Hospital (PRTH), located at the Puerto Rico Medical Center, is the only acute care center in PR and the Caribbean and was one of the few hospitals that remained operational during the emergency.

There is limited research addressing outcomes associated with falls after a natural disaster. Therefore, we aimed to compare the sociodemographic characteristics, clinical profile, hospital course, and cost of services for patients admitted to the PRTH for traumatic falls after Hurricane Maria with a control period. This study will help elucidate the health consequences associated with falls and improve healthcare preparedness and interventions in the context of hazardous environmental conditions, such as those produced by a powerful hurricane.

## Methods

A retrospective cohort study was conducted using patient’s data from the PRTH, a state designated level 1 trauma center in Puerto Rico. Patient’s admission and discharge data was retrieved using the Puerto Rico Trauma Registry, which is part of the US National Trauma Registry System. The study aimed to evaluate clinical, hospital course and outcomes of patients admitted to the PRTH before and after the hit of Hurricane Maria.

### Patient population

A retrospective cohort of patients ≥18 years of age were followed during a four-month period. Two cohorts were identified: eighty-five patients admitted for falls mechanism of trauma from September 20, 2017 through January 20, 2018 (Post-Maria cohort) and two-hundred and eighty-one patients admitted for falls mechanism of trauma from the same 4-months periods (September 20through January 20) from the years 2014, 2015 & 2016 (Pre-Maria cohort). We evaluated the likelihood of multiple hospitalizations associated with the same injury in order to include only the patient’s first admission. However, we did not identify any cases with multiple hospitalizations during the periods under evaluation.

### Variables

We evaluated data from the following domains: sociodemographic, clinical and hospital course factors, costs, and in-hospital mortality.

For the sociodemographic domain, we considered the following parameters: sex, age, health insurance, and work-related admissions. The International Classification of Diseases, 9th & 10th revision (ICD-9 & ICD-10), was used to identify injuries associated with falls. The following codifications were identified in our database: E880–888, W00-W19.

As to trauma prognosis, we used the Injury Severity Score (ISS), which was evaluated as a continuous variable and by categories, and the Glasgow Coma Scale (GCS). We also assessed the Abbreviated Injury Scale (AIS) that classifies injuries based on six body regions (head & neck, face, chest, abdomen, extremity, and external), by using a six-point scale. The AIS aims to quantify the ‘threat to life’, but it does not intend to be a measure of injury severity (Copes et al. 1990). An AIS value ≥1 was defined as an injury in the specific body region. We also classified the AIS score in three main categories: none: AIS = 0, mild/moderate: AIS 1–2, serious through unsurvivable: AIS 3–6.

The most common trauma fractures found in our database were also evaluated: skull, ribcage, spine, and upper/lower limb. A sub-analysis of fall altitude among those subjects whose data was available (*n* = 116) was also evaluated and categorized as fall < 1 m (3.3 ft), 1–6 m (3.3–19.7 ft), and > 6 m (19.7 ft). Time parameters measured included the days in the intensive care unit (ICU), days of mechanical ventilation (MV), and hospital length of stay (LOS). Total hospitalization costs (in US dollars) were also compared between the cohorts. Finally, in-hospital mortality was defined as the rate of death during the hospital stay.

### Statistical analysis

Descriptive statistics was done using the mean, standard deviation (sd), median, and interquartile range for continuous data, while categorical variables were described as frequencies and percentages. The primary independent variable was the study period (Pre-Maria and Post-Maria). Comparisons between the cohorts with the other study covariates were performed using the Wilcoxon rank-sum test for continuous data, whereas the Pearson’s chi-square test statistic or Fisher’s exact test were used for categorical variables, as appropriate.

To complete the analysis, crude and adjusted unconditional logistical regression was calculated taking the study period as the outcome variable. For each variable evaluated in the model, the odds ratio (OR) and 95% confidence interval (CI) were computed. Finally, the multivariate model was further adjusted for ISS. Once the model was generated, the fit conditions were properly addressed.

Statistical significance for bivariate and multivariate analyses was set at *p* < 0.05. The statistical software STATA version 14 (STATA Corp, College Station, TX, USA) was used to perform the analysis. Approval for this study was obtained from the Institutional Review Board of the Medical Sciences Campus of the University of Puerto Rico.

## Results

A total of 85 subjects were admitted to the PRTH for traumatic falls post-Hurricane Maria; whereas 281 were admitted for traumatic falls in the pre-Maria cohort: 105 in 2014, 101 in 2015 and 75 subjects in 2016. Men were the prevalent demographic in both periods (Pre-Maria 82.9% vs Post-Maria 84.7%; *p* = 0.70). After the hurricane, a significant increase in traumatic falls among subjects aged 40–64 years (39.2% vs. 50.6%) and a decrease among those aged 18–39 years (16.0% vs. 5.9%) was observed (*p* = 0.03). For those 65 years old and older no significant change was detected, however remained a substantial population. Also, a considerable increase of work-related traumatic falls (3.9 to 9.4%; *p* = 0.05) was reported after the hurricane.

Even though the differences were not statistically significant, a slight increase in the proportion of patients with a GCS between 3 and 8 (10.4% vs. 14.5%; *p* = 0.20), and with an ISS ≥25 was observed after the storm (6.4% vs 9.4%; *p* = 0.30). No significant differences were found between the groups in health care coverage, medical costs, hospital LOS, ICU LOS, mechanical ventilation days and in-hospital mortality. Results for both periods are described in Table 1.

**Table 1:** Sociodemographic profile, hospital length of stay and in-hospital mortality

Variables	Pre-Maria (2014–2016, ***n*** = 281) n (%)	Post-Maria (2017, ***n*** = 85) n (%)	***p***-value
**Sex**			0.70
Male	233 (82.9)	72 (84.7)	
Female	48 (17.1)	13 (15.3)	
**Age in years**			0.03
18–39	45 (16.0)	5 (5.9)	
40–64	110 (39.2)	43 (50.6)	
> 64	126 (44.8)	37 (43.5)	
**Health care coverage**			0.70
Insured	263 (93.6)	73 (94.8)	
Uninsured	18 (6.4)	4 (5.2)	
**Work related**			0.05
Yes	11 (3.9)	8 (9.4)	
No	270 (96.1)	77 (90.6)	
**ISS**			0.30
< 15	174 (62.2)	57 (67.1)	
15–24	88 (31.4)	20 (23.5)	
≥ 15	18 (6.4)	8 (9.4)	
**GCS (*N*** **= 361)**			0.20
13–15	236 (84.6)	69 (84.2)	
9–12	14 (5.0)	1 (1.2)	
3–8	29 (10.4)	12 (14.6)	
**ICU LOS,*days*(*N*** **= 77)**	***n*** **= 56**	***n*** **= 21**	0.99
Median (IQR)	17.5 (24)	21 (13)	
**Hospital LOS,*days***			0.91
Median (IQR)	10 (15)	6 (15)	
**Mechanical ventilation,*days*(*N*** **= 81)**	***n*** **= 59**	***n*** **= 22**	0.78
Median (IQR)	16 (23)	16.5 (16)	
**In-hospital mortality**			0.89
Alive	243 (86.5)	74 (87.1)	
Dead	38 (13.5)	11 (12.9)	
**Costs,*in US dollars***			0.84
Median (IQR)	$19,058.27 ($36,775.01)	$18,623.95 ($40,806.18)	

*ISS* Injury severity score, *GCS* Glasgow Coma Scale, *ICU* Intensive care unit, *IQR* Interquartile range, *LOS* Length of stay

Upon evaluating severity of injury due to the fall, the incidence of face (*p* < 0.01), chest (*p* < 0.01), extremities (*p* = 0.02) and external (*p* = 0.01) injuries increased significantly post-Maria; while head and neck, and abdomen regions showed no significant difference (see Table 2). During both periods, injuries and fractures overall remained constant, although intracranial injuries (14.2% vs. 23.5%; *p* = 0.06) (data not shown) and skull fractures (12.1% vs. 17.7%; *p* = 0.19) showed a slight increase in the time span after the hurricane (Table 3).

**Table 2:** Abbreviated Injury Severity Score (*N* = 366)

Body Region	Pre-Maria (2014–2016, ***n*** = 281) n (%)	Post-Maria (2017, n = 85) n (%)	***p***-value
**Head & Neck**			0.08
Any (≥1) vs. none	109 (38.8)	24 (28.2)	
*Categories*			0.05
Serious injury (≥3)	87 (31.0)	15 (17.7)	
Mild/moderate (1-2)	22 (7.8)	9 (10.6)	
None	172 (61.2)	61 (71.7)	
**Face**			< 0.01
Any (≥1) vs. none	1 (8.0)	14 (16.5)	
*Categories*			< 0.01
Serious injury (≥3)	1 (0.4)	–	
Mild/moderate (1-2)	17 (6.0)	14 (16.5)	
None	263 (93.6)	71 (83.5)	
**Chest**			< 0.01
Any (≥1) vs. none	133 (47.3)	55 (64.7)	
*Categories*			0.02
Serious injury (≥3)	106 (37.7)	46 (54.1)	
Mild/moderate (1-2)	27 (9.6)	9 (10.6)	
None	148 (52.7)	30 (35.3)	
**Abdomen**			0.96
Any (≥1) vs. none	72 (25.6)	22 (25.9)	
*Categories*			0.99
Serious injury (≥3)	23 (8.2)	7 (8.2)	
Mild/moderate (1-2)	49 (17.4)	15 (17.7)	
None	209 (73.4)	63 (74.1)	
**Extremity**			0.02
Any (≥1) vs. none	107 (38.1)	45 (52.9)	
*Categories*			0.05
Serious injury (≥3)	23 (8.2)	11 (12.9)	
Mild/moderate (1-2)	84 (29.9)	34 (40.0)	
None	174 (61.9)	40 (47.1)	
**External**			0.01
Any (≥1) vs. none	21 (7.5)	14 (16.5)	
*Categories*			0.03
Serious injury (≥3)	1 (0.4)	–	
Mild/moderate (1-2)	20 (7.1)	14 (16.5)	
None	260 (92.5)	71 (83.5)

**Table 3:** Trauma fractures profile (*N* = 366)

Characteristic	Pre-Maria (2014–2016, n = 281) n (%)	Post-Maria (2017, n = 85) n (%)	***p***-value
**FRACTURES**			
Skull fracture			0.19
Yes	34 (12.1)	15 (17.7)	
No	247 (87.9)	70 (82.3)	
Ribcage fracture			0.60
Yes	161 (57.3)	46 (54.1)	
No	120 (42.7)	39 (45.9)	
Spine fracture			0.48
Yes	64 (22.8)	16 (18.8)	
No	217 (77.2)	69 (81.2)	
Upper limb fracture			0.18
Yes	68 (24.2)	14 (16.5)	
No	213 (75.8)	71 (83.5)	
Lower limb fracture			0.44
Yes	38 (13.5)	15 (17.6)	
No	243 (86.5)	70 (82.4)	

In sub-analysis, comparing the pre-Maria and post-Maria timeframes, falls from under 1 m of height increased from 36.3 to 45.5% among young and middle-aged individuals. According to GCS stratified by altitude, the most denoting increase was that of patients who presented in a comatose state (GCS 3–8). The frequency of patients with a GCS 3–8 in falls under 1 m increased from 18.2 to 30%, in falls between 1 and 6 m it increased from 0 to 4.5%, and in falls over 6 m it increased from 15.8 to 20%. When evaluating ISS by altitude, subjects who fell from 1 to 6 m had ISS ≥ 15 score increased after the hurricane (25.8% vs. 39.1%). Likewise, in-hospital mortality increased notably in patients that fell from less than 6 m of height, especially in falls under 1 m (9.1% vs. 36.4%) (Table 4).

**Table 4:** Fall altitude by selected group characteristics (*N* = 116)

Characteristic	Fall under 1 m (3.3 ft) ***n*** = 33		Fall 1-6 m (3.3–19.7 ft) ***n*** = 54		Fall over 6 m (19.7 ft) ***n*** = 29	
	2014–16	2017	2014–16	2017	2014–16	2017
Age						
< 65	8 (36.3)	5 (45.5)	19 (61.3)	13 (56.5)	13 (68.4)	6 (60.0)
≥ 65	14 (63.6)	6 (54.5)	12 (38.7)	10 (43.5)	6 (31.6)	4 (40.0)
GCS (*N* = 114)						
13–15	18 (81.8)	7 (70.0)	30 (96.8)	21 (95.5)	13 (68.4)	8 (80.0)
9–12	–	–	1 (3.2)	–	3 (15.8)	–
3–8	4 (18.2)	3 (30.0)	–	1 (4.5)	3 (15.8)	2 (20.0)
ISS						
< 15	14 (63.6)	7 (64.6)	23 (74.2)	14 (60.9)	9 (47.4)	6 (60.0)
≥ 15	8 (36.4)	4 (36.4)	8 (25.8)	9 (39.1)	10 (52.6)	4 (40.0)
In-hospital mortality						
Alive	20 (90.9)	7 (63.6)	28 (90.3)	19 (82.6)	16 (84.2)	9 (90.0)
Dead	2 (9.1)	4 (36.4)	3 (9.7)	4 (17.4)	3 (15.8)	1 (10.0)

*GCS* Glasgow Coma Scale, *ISS* Injury severity score

In bivariate analysis, during the post-Maria period, trauma patients with fall-related injuries were 3.05 times more likely to be ≥40 years of age (95% CI: 1.17–7.95). Moreover, these patients were also less critically ill as per the evaluation of their ISS score. Trauma patients with fall-related injuries were 2.55 times more likely to present with a work related traumatic injury. In addition, a marginal 19% lower odds of having an ISS between 15 and 75 was observed in the Post-Maria cohort, even though not statistically significant. In multivariate analysis, after adjusting for ISS, age ≥ 40 years [OR (95%CI): 3.20 (1.22–8.40)] and work related injuries [OR (95%CI): 6.64 (1.01–6.91)] were strongly associated with the Post-Maria period (Table 5).

**Table 5:** Bivariate and multivariate analysis for the post-Maria period

Characteristic	Crude OR (95% CI)	Adjusted OR (95% CI)
Age, *years*		
≥ 40	3.05 (1.17–7.95)	3.20 (1.22–8.40)
Work related		
Yes	2.55 (1.00–6.56)	6.64 (1.01–6.91)
ISS		
15–75	0.81 (0.48–1.35)	–

*OR* Odds Ratio, *CI* Confidence Interval, *ISS* Injury Severity Score

## Discussion

The hazardous conditions after a hurricane increase the risk for trauma and change injury patterns in the population. Specifically, falls increase in middle-aged men, although this type of injury is typically seen on women and geriatric population (Malilay et al. 2014; Uscher-Pines et al. 2009). Studies have found a high incidence of falls, while no change in admissions post-hurricane (Curran et al. 2017). Our study evidenced an increase in admissions for traumatic falls in the middle-aged population, even though older-adults remained an important demographic. This demonstrates that these age groups should be the focus of attention after any major disaster. Extreme weather events place working-age adults at further risk of injury, as residents and relief workers go out to remove debris, open access to transit, and restore power (Marshall et al. 2018; Rusiecki et al. 2014; Seil 2016). Moreover, it is well known that the elderly are susceptible to suffer falls, which makes them prone to suffering a fall when environmental conditions are worse or when they have to perform duties that do not fit their capabilities (Bergen et al. 2014; Hefny et al. 2016; Rubenstein 2006; Florence et al. 2018; Alizo et al. 2018). In addition, this population usually refuses to evacuate their homes and need rapid and prolonged assistance (Uscher-Pines et al. 2009; Curran et al. 2017; Adams et al. 2011; Al-Rousan et al. 2014). The experience with Hurricanes Sandy and Katrina was that the victims were most likely the geriatric population (Wahl et al. 2009; Lin et al. 2002; Seil 2016). According to the US Census, the elderly population is rapidly increasing, especially in PR, due to the high emigration of younger individuals, low birth rate, and longer life expectancy (United States Census Bureau 2018). Understanding the needs of this increasing population will be paramount for health services planning.

Falls constitute a problem to society and public health, representing an increased use of healthcare services and impacting the quality of life and independence of the injured, more so after a major natural disaster (Hefny et al. 2016; Florence et al. 2018). After an extreme weather event, the entire population is exposed to fall-related injuries. Even though most recovery-related hard work is carried out by men (Malilay et al. 2014; Uscher-Pines et al. 2009), our study found no significant difference in gender. Men remained the prevalent demographic for trauma, even though under normal conditions falls are most common in older women (Adams et al. 2011; Berry and Miller 2008; Rubenstein 2006; Alamgir et al. 2015; Florence et al. 2018; Lee et al. 2013; Santos-Burgoa et al. 2018; Cruz-Cano and Mead 2019). However, we do report that work-related traumatic falls increased after Hurricane Maria. Similarly, it is well known in the literature that those working in assistance, rebuilding, and cleaning are at increased risk for injury (Malilay et al. 2014; Uscher-Pines et al. 2009; Curran et al. 2017; Berry and Miller 2008; Rusiecki et al. 2014).

Results of this study show an increase in the proportion of patients who presented with poor prognosis, higher ISS, and lower GCS after the hurricane. Similarly, post-Hurricane Katrina hospital admissions resulted in patients with higher acuity and ISS, lower initial GCS and blunt trauma (Wahl et al. 2009). Post-hurricane hazardous conditions influence the gravity of the trauma, as time of arrival to the PRTH increased, power was interrupted frequently, and resources and personnel were limited. However, our data revealed no difference in in-hospital mortality post-hurricane. Numerous research studies state that falls are characterized by high medical and social costs, and that after a devastating event, those costs are likely to increase (Florence et al. 2018; Gevitz et al. 2017). Nonetheless, this study found no significant difference in medical costs after the hurricane.

Fall-related injuries after a natural disaster have not been described previously, yet it is known that common injuries after a fall include bone fractures, head injuries, wounds, bruises, and sprains (Berry and Miller 2008; Frasqueri-Quintana et al. 2019; Burns and Kakara 2018; Bergen et al. 2014; Hefny et al. 2016). In the post-hurricane period, severity of injury increased significantly in fall-related injuries to the face, chest, extremities and external (skin and connective tissue), and a slight decrease in injuries to head and neck. Other studies evaluating multiple mechanisms of injury after a major disaster have found similar results. A study evaluating visits to an emergency room post-Hurricane Maria in the southern area of Puerto Rico reported that the most common types of injuries observed were abrasions, lacerations, cuts, sprains, head injuries, and concussions (Frasqueri-Quintana et al. 2019).

Fractures to the extremities after a fall are also well characterized and can vary depending on the fall height. In our study, of the patients with fall height information, post-hurricane falls from under one meter increased among individuals < 65 years and decreased among the elder. In falls up to 6 m high an increase was observed in those 65 years and older. In the post-hurricane period of our study, more patients were admitted with a low GCS related to high-altitude falls and even falls from own feet. Of the patients with an ISS ≥15, more admissions resulted of falls from 1 to 6 m and fewer patients sustained higher falls after Hurricane Maria. In-hospital mortality frequency increased notably in falls from under one meter height, which include falls from own feet.

Limitations in this study include its retrospective nature; fall altitude information for all patients was not available. For multivariate analysis purposes, GCS cutoff was set at < 15 and ≥ 15 due to insufficient number of subjects for use of the conventional cutoff of 15–13, 12–9 and 8–3. Additionally, the sample was limited to the trauma patient’s data from the PRTH because this is the only hospital specialized in treating acute traumatic injuries in Puerto Rico. Moreover, this is one of the few studies evaluating traumatic injuries after a natural disaster. Future research should evaluate multiple trauma centers and include patient follow-up data to help advance the understanding and prevention of fall-related injuries after a natural disaster.

## Conclusions

Etched in the history books, Hurricane Maria will always represent one of the worst natural disasters Puerto Rico has ever faced and will most likely not be the last one. The importance of such tragic events relies on our ability to learn from them and adapt in order to avoid the same pitfalls in the future. As the highest-level Trauma Center available in Puerto Rico and the Caribbean, the PRTH must remain updated on the current traumatic epidemiological trends in order to better guide its efforts in the face of disaster. This study is an attempt to provide a comprehensive description of traumatic falls after a natural disaster and to evaluate if there was a change in the previously known epidemiological trend regarding this mechanism of injury. As it turns out, there was a shift in the known paradigm since fall-related injuries increased among middle-aged individuals after the hurricane, compared to the well-known predominant group associated with falls which is the elderly.

It is our hope that the analysis provided can be used to develop cost-effective prevention strategies focused in the vulnerable populations to reduce fall-related injuries that would lead to a decline in morbidity and mortality. Falls, in most instances, are considered a preventable injury and being aware of environmental hazards can reduce the risk of falling. The understanding gathered with this information will help guide new efforts to improve all aspects of health care from prevention to the rehabilitation process in future disasters.

## Data Availability

The data that supports the findings of this study is available from the Puerto Rico Trauma Hospital Data Registry but restrictions apply to its availability. Data is however available from the authors upon reasonable request and with permission of the Puerto Rico Trauma Hospital.

## Abbreviations

GCS: Glasgow Coma Scale
ISS: Injury Severity Score
ICU: Intensive Care Unit
PR: Puerto Rico
US: United States
PRTH: Puerto Rico Trauma Hospital
ICD-10: International Classification of Diseases, 10th Revision
AIS: Abbreviated Injury Score
MV: Mechanical Ventilation
LOS: Length of stay in day units
sd: Standard Deviation
OR: Odds Ratio
CI: Confidence Interval
